# Human Papillomaviruses Activate and Recruit SMC1 Cohesin Proteins for the Differentiation-Dependent Life Cycle through Association with CTCF Insulators

**DOI:** 10.1371/journal.ppat.1004763

**Published:** 2015-04-13

**Authors:** Kavi Mehta, Vignesh Gunasekharan, Ayano Satsuka, Laimonis A. Laimins

**Affiliations:** Department of Microbiology-Immunology, Feinberg School of Medicine, Northwestern University, Chicago, Illinois, United States of America; National Institute of Allergy and Infectious Diseases, National Institutes of Health, UNITED STATES

## Abstract

Human papillomaviruses infect stratified epithelia and link their productive life cycle to the differentiation state of the host cell. Productive viral replication or amplification is restricted to highly differentiated suprabasal cells and is dependent on the activation of the ATM DNA damage pathway. The ATM pathway has three arms that can act independently of one another. One arm is centered on p53, another on CHK2 and a third on SMC1/NBS1 proteins. A role for CHK2 in HPV genome amplification has been demonstrated but it was unclear what other factors provided important activities. The cohesin protein, SMC1, is necessary for sister chromatid association prior to mitosis. In addition the phosphorylated form of SMC1 plays a critical role together with NBS1 in the ATM DNA damage response. In normal cells, SMC1 becomes phosphorylated in response to radiation, however, in HPV positive cells our studies demonstrate that it is constitutively activated. Furthermore, pSMC1 is found localized in distinct nuclear foci in complexes with γ-H2AX, and CHK2 and bound to HPV DNA. Importantly, knockdown of SMC1 blocks differentiation-dependent genome amplification. pSMC1 forms complexes with the insulator transcription factor CTCF and our studies show that these factors bind to conserved sequence motifs in the L2 late region of HPV 31. Similar motifs are found in most HPV types. Knockdown of CTCF with shRNAs blocks genome amplification and mutation of the CTCF binding motifs in the L2 open reading frame inhibits stable maintenance of viral episomes in undifferentiated cells as well as amplification of genomes upon differentiation. These findings suggest a model in which SMC1 factors are constitutively activated in HPV positive cells and recruited to viral genomes through complex formation with CTCF to facilitate genome amplification. Our findings identify both SMC1 and CTCF as critical regulators of the differentiation-dependent life cycle of high-risk human papillomaviruses.

## Author Summary

Over 120 types of human papillomavirus (HPV) have been identified, and approximately one-third of these infect epithelial cells of the genital mucosa. Infection by a subset of HPV types is responsible for the development of cervical and other anogenital cancers. The infectious life cycle of HPV is dependent on differentiation of the host epithelial cell, with viral genome amplification and virion production restricted to differentiated suprabasal cells. While normal keratinocytes exit the cell cycle upon differentiation, HPV positive suprabasal cells are able to re-enter S-phase to mediate productive replication. HPV induces an ATM-dependent DNA damage response that is essential for viral genome amplification in differentiating cells. In this study we demonstrate that a protein that mediates sister chromatid association prior to mitosis, SMC1, plays a critical role in the differentiation-dependent replication of HPV through the recruitment of DNA damage proteins to viral genomes. SMC1 binds specifically to CTCF binding sites in the late region of HPV through association with the DNA insulator protein CTCF. Knockdown of either SMC1 or CTCF abrogates viral genome amplification. Further, mutation of CTCF sites in the late region of the HPV genome results in loss of both episomal maintenance and the ability for SMC-1 and CTCF to interact with the genome. Our findings identify an important regulatory mechanism by which HPV controls replication during the productive phase of the life cycle, and this can lead to new targets for the development of therapeutics to treat HPV induced infections.

## Introduction

Human papillomaviruses are the causative agents of cervical and other anogenital malignancies. HPV-16, 18, 31 along with at least ten other types are referred to as high-risk as they are associated with the development of genital cancers[1] [2]. These high-risk viruses infect squamous epithelial cells in the genital tract and link their productive life cycles to differentiation. HPVs infect cells in the basal layers of stratified epithelia and establish their genomes as nuclear episomes at about 50 to 100 copies per cell [3]. In infected basal cells, viral genomes are replicated along with cellular DNA and distributed equally to the two daughter cells[4]. While one daughter cell remains in the basal layer, the other migrates away and undergoes differentiation in suprabasal layers. HPVs do not encode their own polymerases and rely on cellular enzymes to replicate their genomes. Normally, daughter cells that migrate from the basal layer exit the cell cycle, however, in HPV infections these cells remain active in the cell cycle and re-enter S/G2 in suprabasal layers to productively replicate their genomes in a process called amplification[5] [6]. Amplification is coincident with activation of the late viral promoter and synthesis of capsid virions [4] [7] [8]. Two processes that regulate the differentiation-dependent viral late phase include maintenance of cell cycle competence in suprabasal cells and activation of the ATM DNA damage response.

In normal cells, the Ataxia-Telangiectasia Mutated (ATM) DNA damage pathway mediates the repair of double strand breaks. A trimeric MRN complex consisting of MRE11, RAD50, and NBS1 first recognizes double strand breaks induced by irradiation or chemicals[9]. The MRN complex recruits ATM proteins to sites of double strand breaks resulting in its activation through autophosphorylation [10]. The ATM kinase then activates a series of downstream effectors that can be grouped into three pathways: p53/p21, CHK2/CDC25 and NBS1/SMC1 [10] [11] [12]. Phosphorylation of these proteins results in cell cycle checkpoint arrest in G1, G2, or S depending on which pathway is utilized. Induction of p53 phosphorylation leads to G1/S arrest while activation of CHK2/CDC25 as well as NBS1/SMC1 leads to G2/S arrest [11] [13] [14]. ATM also induces the phosphorylation of a specific type of histone, called γ -H2AX, that is recruited to regions that are tens of kilobases in size that flank sites of double strand breaks [15] [16]. While activation of the ATM pathway is important for HPV genome amplification, it is not clear which set of the ATM factors provide necessary functions.

SMC1 proteins are members of the Structural Maintenance of Chromosomes (SMC) family of proteins that play critical roles in organizing and stabilizing chromosomal segregation during mitosis. SMC1 forms a heterodimeric cohesin complex with SMC-3 that encircles and mediates sister chromatid cohesion for proper chromosome segregation[17]. Two other members of this family, SMC2 and SMC4, associate to form the condensin complex that regulates chromosome condensation[18]. In addition to its role in chromosome segregation, SMC1 can be phosphorylated by ATM and plays a central role in mediating G2/M checkpoint arrest as well as homologous recombination repair [12] [14]. As shown by Yazdi et al. SMC1 is part of the NBS1 arm of the ATM pathway, which is distinct and acts independently from the CHK2/CDC25 pathway[12].

Previous studies have shown that ATM and CHK2 are important regulators of HPV genome amplification[5] [19] [20] [21]. In the present study, we investigated if SMC1 is important for HPV genome amplification in differentiating cells. Our studies demonstrate that HPV proteins induce the constitutive activation and phosphorylation of SMC1 (S957) in both undifferentiated and differentiated cells. In HPV positive cells, active, phosphorylated SMC1 proteins localize to foci or puncta in the nucleus in complexes with other members of the ATM pathway. We further demonstrate that SMC1 is recruited to HPV genomes through association with cellular CTCF insulator proteins. Importantly, inhibition of SMC1 or CTCF activity through shRNA knockdowns impairs differentiation-dependent genome amplification. This study identifies SMC1 and CTCF as critical components of the ATM pathway that are important for differentiation-dependent genome amplification.

## Results

**Fig 1:**
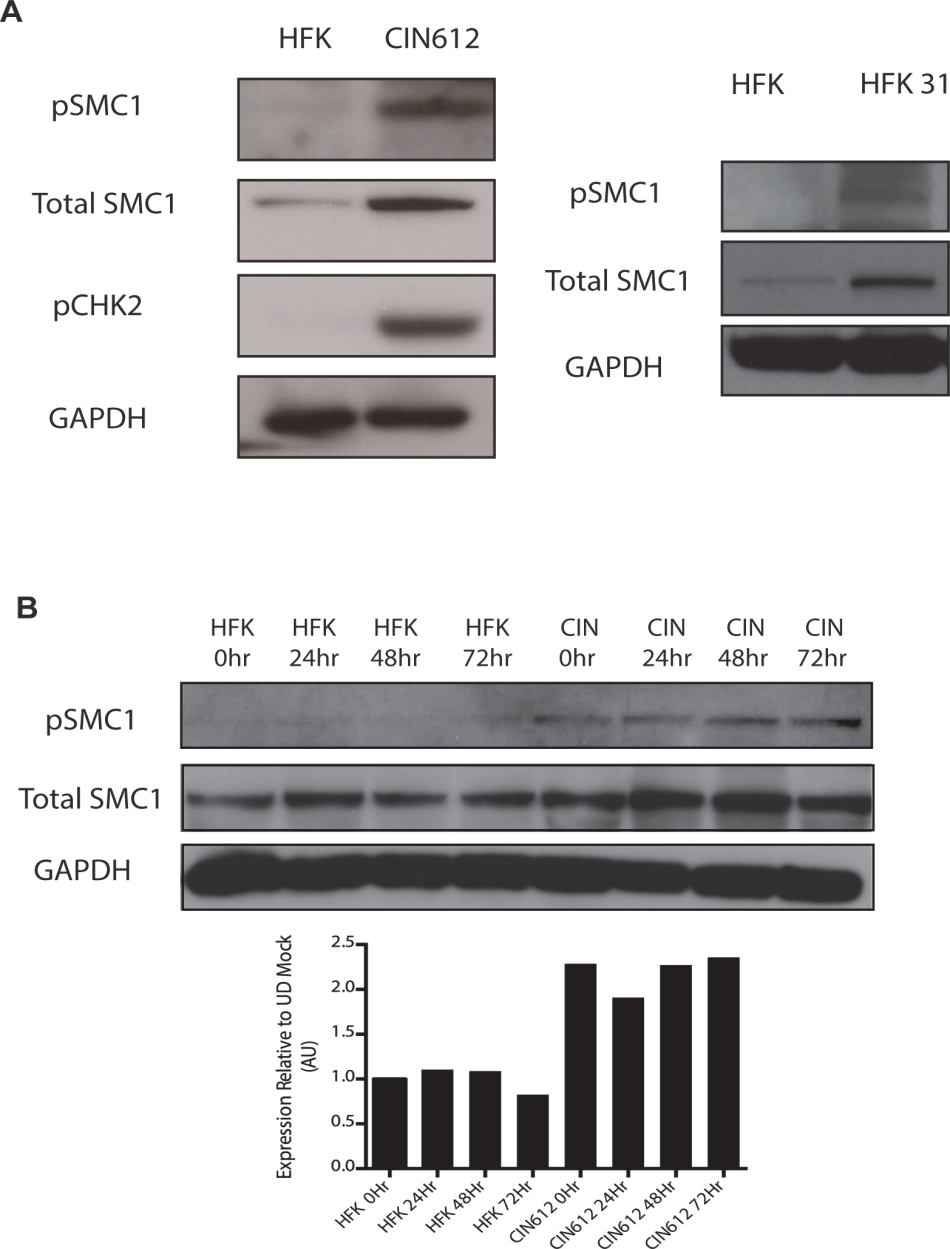
Levels of total SMC1 and pSMC1 are increased in HPV positive cells and remain high upon calcium-induced differentiation. A). Whole cell extracts were isolated from undifferentiated normal human foreskin keratinocytes (HFK) and HPV 31 positive CIN 612 cells, and HFKs stably transfected to maintain HPV-31 genomes grown in monolayer culture and examined by Western blot analysis with antibodies to total SMC1, pSMC1 and pCHK2. GAPDH served as a loading control. B). CIN612 cells and HFKs were induced to differentiate by addition of high calcium media and cell extracts harvested at 0, 24, 48 and 72 hours. Western blot analysis was performed with antibodies against total SMC1 and pSMC1. GAPDH served as a loading control. Quantitation of band intensity revealed an approximate 2-fold, differentiation-independent increase of SMC1 protein levels in HPV-positive cells.

To investigate if SMC1 structural maintenance proteins play any role in the life cycle of HPV, we first examined if the levels of total and phosphorylated SMC1 are altered between normal and HPV positive keratinocytes. Western blot analysis demonstrated that levels of total, and phosphorylated SMC1, are increased by approximately two fold in HPV positive cells as compared to normal keratinocytes (Fig 1A). Similar activation of SMC1 is also seen in HPV-18 positive cell lines. Consistent with previous observations, the levels of active pCHK2 are also significantly increased in HPV positive cells (Fig 1A)[19]. It was next important to determine if epithelial differentiation alters the levels of total or phosphorylated SMC1 proteins. For this analysis we used calcium induced differentiation of epithelial cells as this allowed for the isolation of uniformly differentiated populations of cells. HPV amplification begins about 48 hours after addition of calcium and plateaus at approximately 72 to 96 hours[19]. Our studies show that the levels of both total SMC1 and pSMC1 in HPV positive cells remain elevated at similar levels throughout differentiation in contrast to normal keratinocytes (Fig 1B). HPV E7 oncoproteins are able to induce pSMC1 in the absence of complete replicating viral genomes (S1 Fig).

**Fig 2:**
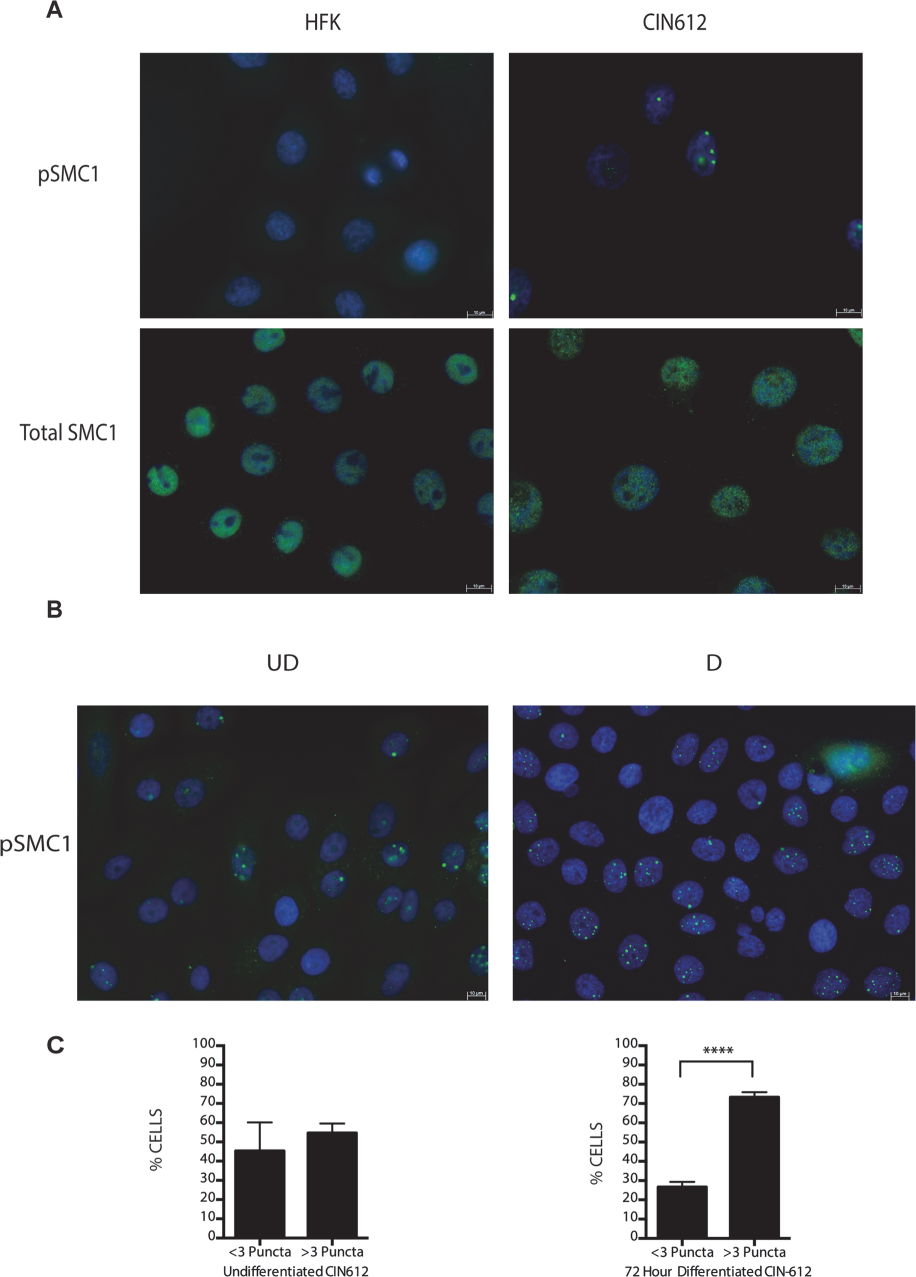
pSMC1 is localized to nuclear foci in HPV positive cells that become more numerous upon differentiation. A) Immunofluorescence analysis of undifferentiated HFKs and CIN612 cells for pSMC1 and total SMC1 demonstrates localization to the nucleus in HPV positive cells. Green indicates either pSMC1 (s957) or total SMC1 as indicated. Blue represents nuclear DAPI staining. B). Immunofluorescence of undifferentiated and 72-hour calcium-induced differentiated CIN612 cells identifies large discrete foci of pSMC1 in HPV positive cells. The left panel represents undifferentiated cells while the right panel represents differentiated cells. Green identifies either pSMC1 (s957) or total SMC1 as indicated. Blue represents nuclear DAPI staining. C). The number of pSMC1 foci greater than 3 per nucleus increases upon differentiation of HPV 31 positive cells. The number of pSMC1 nuclear foci in 559 individual undifferentiated and 569 differentiated cells obtained from 3 separate experiments was determined. The graph demonstrates the percentage of total nuclei with greater than 3 foci per nucleus increasing to 80% of cells upon differentiation in high calcium media for 72 hours. A standard students t-test was used to determine statistical significance of <.0001.

**Fig 3:**
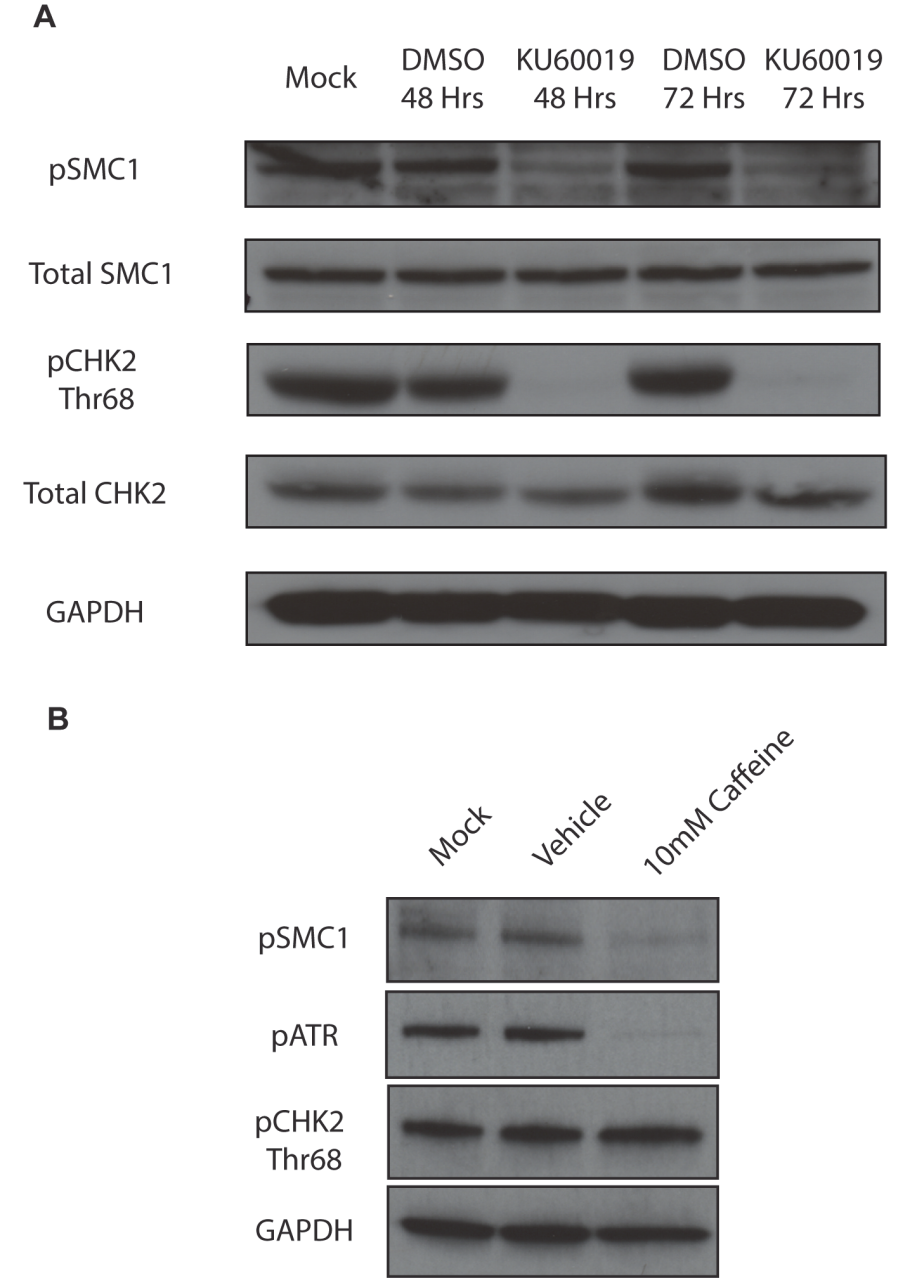
SMC1 is phosphorylated by both ATM and ATR. A). ATM inhibitor, KU60019 inhibits CHK2 phosphorylation, but only partially reduces SMC1 phosphorylation in HPV positive cells. HPV 31 positive CIN 612 cells were treated with the ATM kinase inhibitor KU60019 (10uM) in DMSO or DMSO alone for 48 and 72 hours and cell extracts examined by Western blot analysis. pCHK2 levels are inhibited by KU60019 while pSMC1 levels are only partially reduced suggesting another kinase such as ATR is involved in phosphorylation. GAPDH is included as a loading control. B) DNA damage response inhibitor Caffeine (10mM) inhibits both pSMC1 and pATR after 48 hours of treatment.

Following DNA damage, a subset of SMC1 proteins become phosphorylated and associate with regions adjacent to double stranded breaks[22],[23],[24],[25]. We next investigated if HPV alters the localization of SMC1 proteins. For this analysis, we examined HPV positive CIN 612 cells as well as normal keratinocytes by immunofluoresence to observe localization of SMC1 and pSMC1. In normal keratinocytes, total SMC1 proteins are distributed uniformly throughout the nucleus but few pSMC1 foci are observed (Fig 2A). In contrast, in HPV positive cells, high levels of pSMC1 positive foci are observed that are localized to the nucleus (Fig 2A). Consistent with previous observations, pCHK2 is found only in HPV positive cells and is localized to foci in the nucleus [20]. Upon differentiation of HPV positive cells, an increase in the number and size of nuclear foci containing pSMC1 is seen (Fig 3B) while no such foci were seen in normal keratinocytes. Specifically the number of nuclei with 3 or more pSMC1 foci increases significantly upon differentiation to approximately 80% of cells (Fig 2C).

We next investigated if ATM or another kinase was responsible for the phosphorylation of SMC1. For this analysis we treated cells with the specific ATM kinase inhibitor, KU60019, and screened for levels of pSMC1 as well as pCHK2, a known target of the ATM kinase, by Western blot analysis[26]. Treatment with KU60019 was found to completely abolish phosphorylation of CHK2, however, it only partially inhibited phosphorylation of SMC1 (Fig 3A). This indicates that phosphorylation of SMC1 is partially dependent upon ATM kinase activity as well as an additional kinase which is most likely ATR. We next treated cells with the DNA damage inhibitor, caffeine, and found that it significantly reduces levels of pSMC1 but not pCHK2 (Fig 3B). We conclude that both ATM and ATR kinases target SMC1 in HPV positive cells and is consistent with previous reports examining normal cells following DNA damage[12], [14], [27].

**Fig 4:**
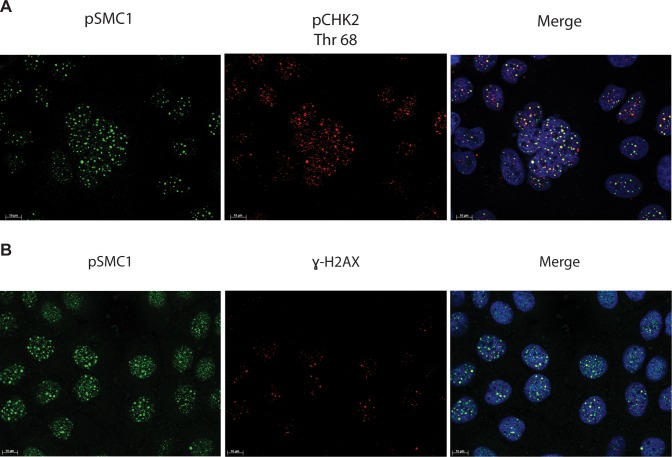
pSMC1 and pCHK2 as well as pSMC1 and γ-H2AX co-localize in distinct foci in nuclei of HPV positive cells following differentiation. CIN 612 cells were induced to differentiate by addition of high calcium media for 72 hours and examined by immunofluorescence for co-localization of A). pSMC1 and pCHK2; B), pSMC1 and γ-H2AX using the corresponding antibodies. Red signal identifies pCHK2 or γ-H2AX in top or bottom panels as shown. Green identifies pSMC1 (s957). Blue represents nuclear DAPI staining. Yellow signal in merged views demonstrates co-localization. The antibody to pCHK2 Thr68 (Cell Signalling) may be responsive to DNA damage factors in general.

**Fig 5:**
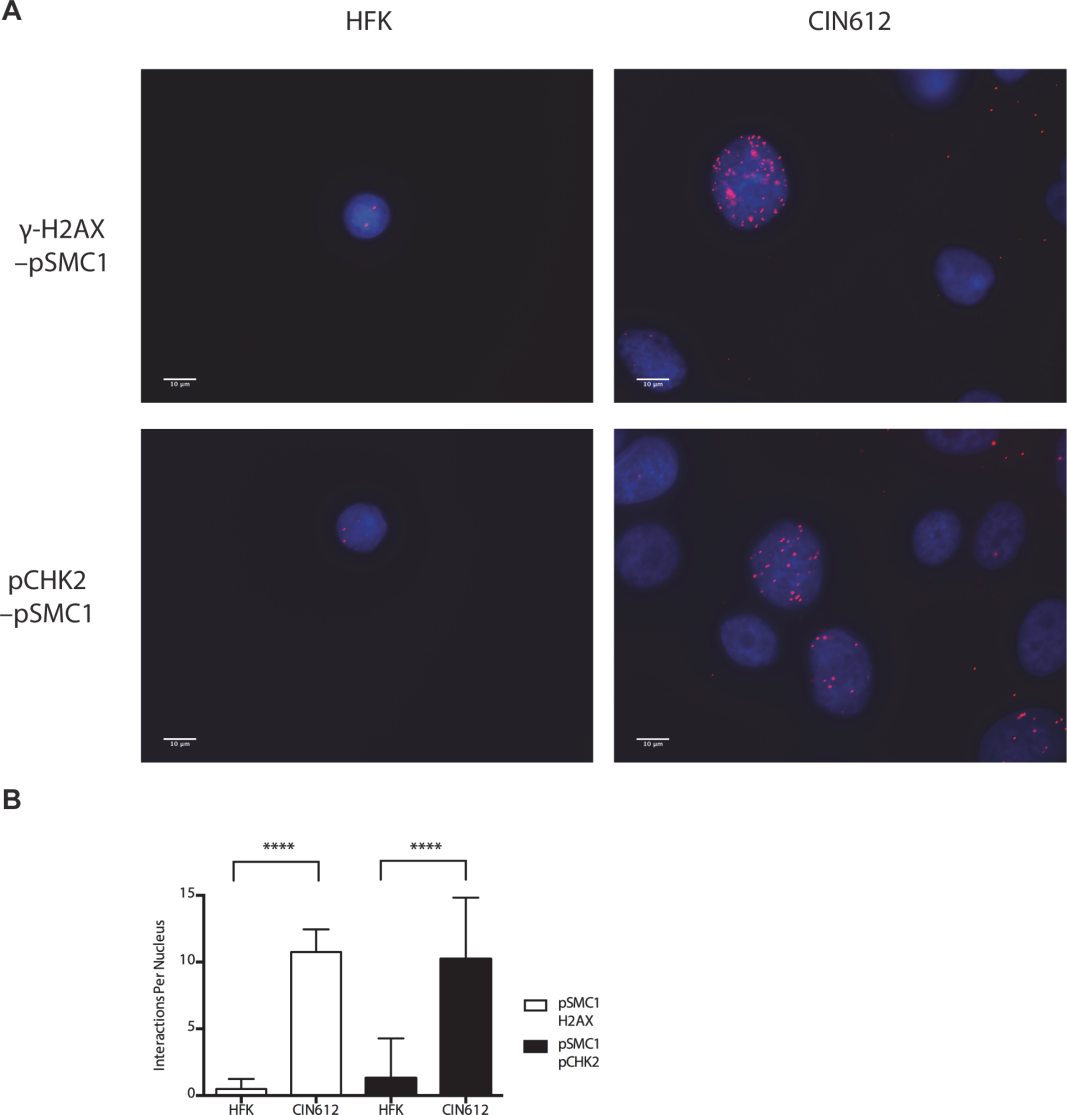
On-slide co-immunoprecipitations for pSMC1 in HFKs and HPV positive cells shows a direct interaction between pSMC1 and γ-H2AX as well as pSMC1 and pCHK2 in HPV positive cells but not HFKs. A). HFKs and CIN 612 cells grown in monolayer cultures were treated with 4% Methanol-free formaldehyde in PBS. Cells were permeabilized in PBS +. 1% Triton X-100 (PBT) and blocked in Normal Goat Serum (NGS+T) (Invitrogen) with. 1% Triton X-100. Primary antibodies were added to the NGS+T and incubated for one hour. Secondary antibodies, covalently linked with plus/minus oligos (OLINK DuoLink, Upsalla Sweden) were added and incubated for an additional 45 minutes. Ligations, and polymerase amplifications, and washes were performed as per the manufacturer’s instructions. All incubations were performed in a humidity chamber. The appearance of red foci indicates complex formation in distinct nuclear puncta. B). Quantitation of number of red interaction foci from on-slide co-immunoprecipitation analyses. For the interaction between pSMC1 and γ-H2AX 132 HFKs and 71 CIN612 cells were examined. For the interaction between pSMC1 and pCHK2 187 HFKs and 92 CIN612 cells were examined. Foci counts are from three individual experiments. Interactions of these factors with pSMC1 in HPV positive cells is statistically significant as indicated by the students t-test, p≤.0001.

In HPV positive cells, members of the ATM pathway such as pCHK2 and γ-H2AX are recruited to nuclear foci that also contain viral genomes[20]. To determine if pSMC1 is co-localized to ATM-positive foci, dual immunofluoresence assays were performed on HPV positive cells. We observed both pCHK2 and γ-H2AX to co-localize to the same foci as pSMC1 in differentiated HPV positive cells (Fig 4). It was next important to determine if pCHK2 and γ-H2AX formed complexes with pSMC1 or whether they merely localized to similar regions in the nucleus. For this analysis we used proximity ligation methodologies, which is also referred to as on-slide co-immunoprecipitation, as this assay allowed for a demonstration of association as well as the identification of where these complexes are located in cells[28]. The proximity ligation assay involves the use of secondary antibodies that are linked to oligonucleotides with overlapping regions of complementarity. If the proteins are in a complex or close proximity, the oligonucleotides can hybridize and act as primers for polymerase mediating amplification with incorporation of fluorescent nucleotides. The appearance of red signal in immunofluorescence analysis is indicative of complex formation. We performed this analysis using HFKs along with HPV positive cells and found complex formation between γ−H2AX and pSMC1 as well as pCHK2 and pSMC1 in nuclear foci only in HPV positive cells (Fig 5). In normal keratinocytes exposed to the same set of antibodies, only background signals were observed demonstrating that these interactions are specific to HPV positive cells. We conclude that pCHK2, γ-H2AX and pSMC1 associate into complexes at nuclear foci in HPV positive cells.

**Fig 6:**
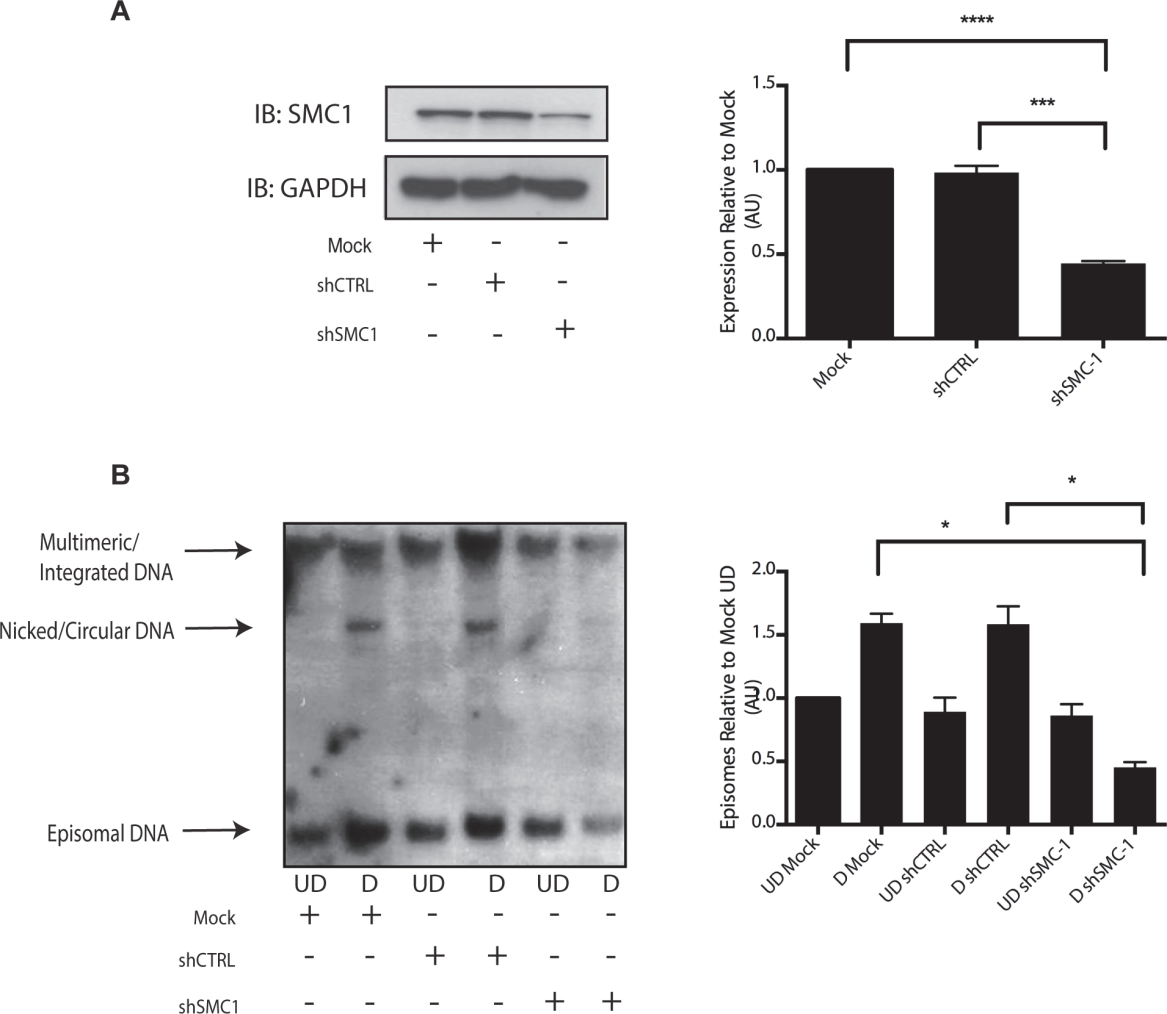
Knockdown of SMC1 with shRNA blocks viral genome amplification. A). CIN612 were infected with lentiviruses encoding shRNAs to SMC1 and after 48 hours total cell extracts were isolated and analyzed by Western blot for levels of SMC1. Quantitation of band intensity revealed an approximate 50% knockdown of SMC1 protein levels on average across three experiments. The knockdown is statistically significant between mock and shSMC and shCTRL and shSMC, p≤.0001, and p≤.001 respectively. B). shRNA knockdown of SMC1 blocks HPV-31 differentiation-dependent viral amplification. Southern blot analysis of CIN 612 cells infected with lentiviruses encoding shRNAs to SMC1 and induced to differentiate in methylcellulose for 48 hours. UD stands for undifferentiated while D stands for differentiated. Total DNA was isolated and examined by Southern analysis for HPV amplification. Similar results were seen in four independent experiments using two different shRNAs as well as the combination of the two. Quantification of band intensity was determined by densitometry using Image J software and represents an average of three independent experiments. SMC knockdown results in a reduction in amplification that is statistically significant, p≤.05

Since our studies indicated that SMC1 proteins form complexes with CHK2, and γ-H2AX at HPV replication centers, it was important to investigate if SMC1 was necessary for HPV amplification. For this analysis we used lentiviral vectors that expressed shRNAs that were effective in partially knocking down levels of SMC1. SMC1 is an essential cellular protein and complete knockdowns are lethal[11]. In our studies, SMC1 levels in HPV 31 positive cells were reduced by approximately 50% through the use of lentivirus shRNA vectors (Fig 6A). HPV positive cells were infected with the recombinant lentiviruses and the reduction in SMC1 levels was confirmed by Western analysis. The cells in which SMC1 had been reduced with shRNAs continued to proliferate at a rate comparable to control cells and exhibited similar percent viability (S2 Fig). The ability to amplify viral genomes upon differentiation was next examined by Southern blot analysis and indicated that reduction of SMC1 levels by approximately 50% is sufficient to inhibit HPV genome amplification (Fig 6B). Similar results were observed in four independent experiments using two different shRNA vectors against SMC1 as well as the combination of the two. Interestingly, reduction of SMC1 levels did not alter the ability of γ-H2AX to form foci in HPV positive cells. We conclude that SMC1 is an important regulator of HPV genome amplification.

**Fig 7:**
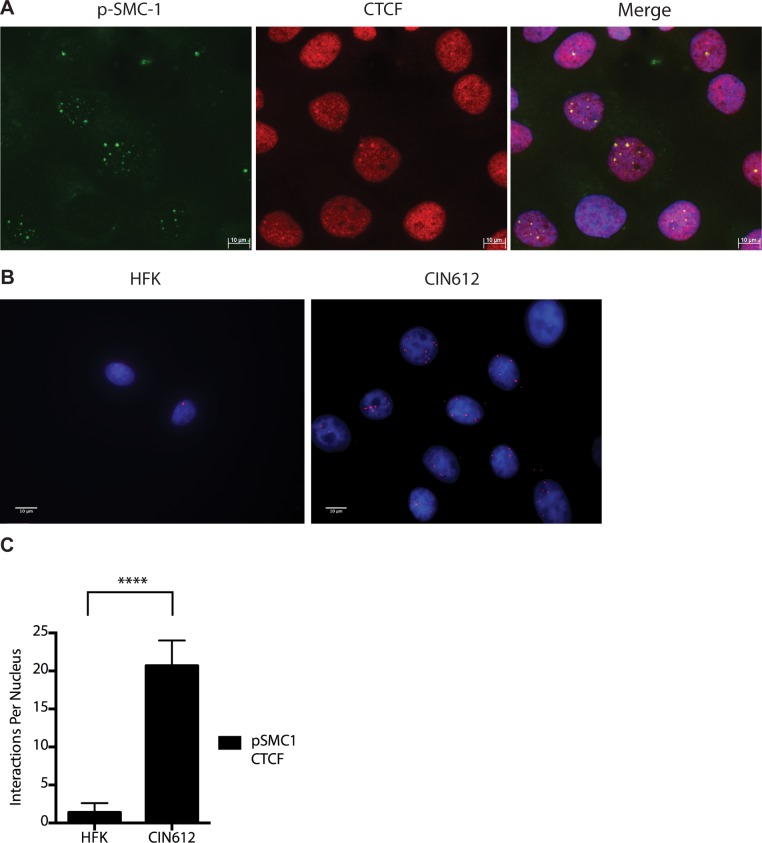
CTCF and SMC1 co-localize to nuclear foci and form complexes. A). Immunofluorescence analysis of CIN 612 cells following calcium induced differentiation for 72 hours. pSMC1 signal is in green, while CTCF proteins are shown in red. The merge is yellow and shows that most CTCF and pSMC1 proteins co-localize in distinct nuclear foci. Numerous CTCF foci are detected in the nuclei of HPV positive cells but only a subset co-localize with pSMC1 while all pSMC1 foci co-localize with CTCF. B).Proximity-ligation assays (on-slide co immunoprecipitation) of pSMC1 and CTCF show complex formation in nuclear foci. C). Quantitation of pSMC1 and CTCF foci in HFK and CIN 612 cells. Foci in 183 HFKs and 107 CIN612 cells from three individual experiments were counted. Interactions of CTCF with pSMC1 in HPV positive cells is statistically significant as indicated by the students t-test, p≤.0001.

As part of the cohesin complex, SMC1 proteins associate broadly with cellular chromosomes to mediate sister chromatid cohesion. SMC1 can also form complexes with CTCF DNA binding proteins to bind to specific sequences [29],[30],[31]. CTCF is a zinc-finger protein that is required for transcriptional insulation as well as DNA looping and chromatin organization [32]. The association of CTCF with SMC1 has been shown to be independent of SMC1’s role in sister chromatid cohesion[33],[34]. Similarly, SMC1 binding to CTCF is not necessary for transcriptional insulation and not all CTCF sites are occupied by SMC1 [29]. We investigated if pSMC1 and CTCF localize to the same nuclear foci in HPV positive cells through co-immunofluorescence analyses. As shown in Fig 7A, pSMC1 and CTCF co-localize to distinct foci in the nuclei of differentiated HPV positive cells that also contain CHK2 and γ-H2AX. Similar CHK2 and γ-H2AX foci in HPV positive cells have been described previously [19, 20]. Numerous CTCF foci are detected in the nuclei of HPV positive cells but only a subset co-localize with pSMC1. In contrast all pSMC1 foci co-localize with CTCF (Fig 7A). Importantly, no pSMC1/CTCF foci were detected in normal keratinocytes demonstrating that such complexes are absent in HFKs. We next investigated if pSMC1 and CTCF formed complexes in these cells through the use of proximity ligation assays. As seen in Fig 7B, complexes are present in HPV positive cells but no signal above background was observed in normal keratinocytes.

**Fig 8:**
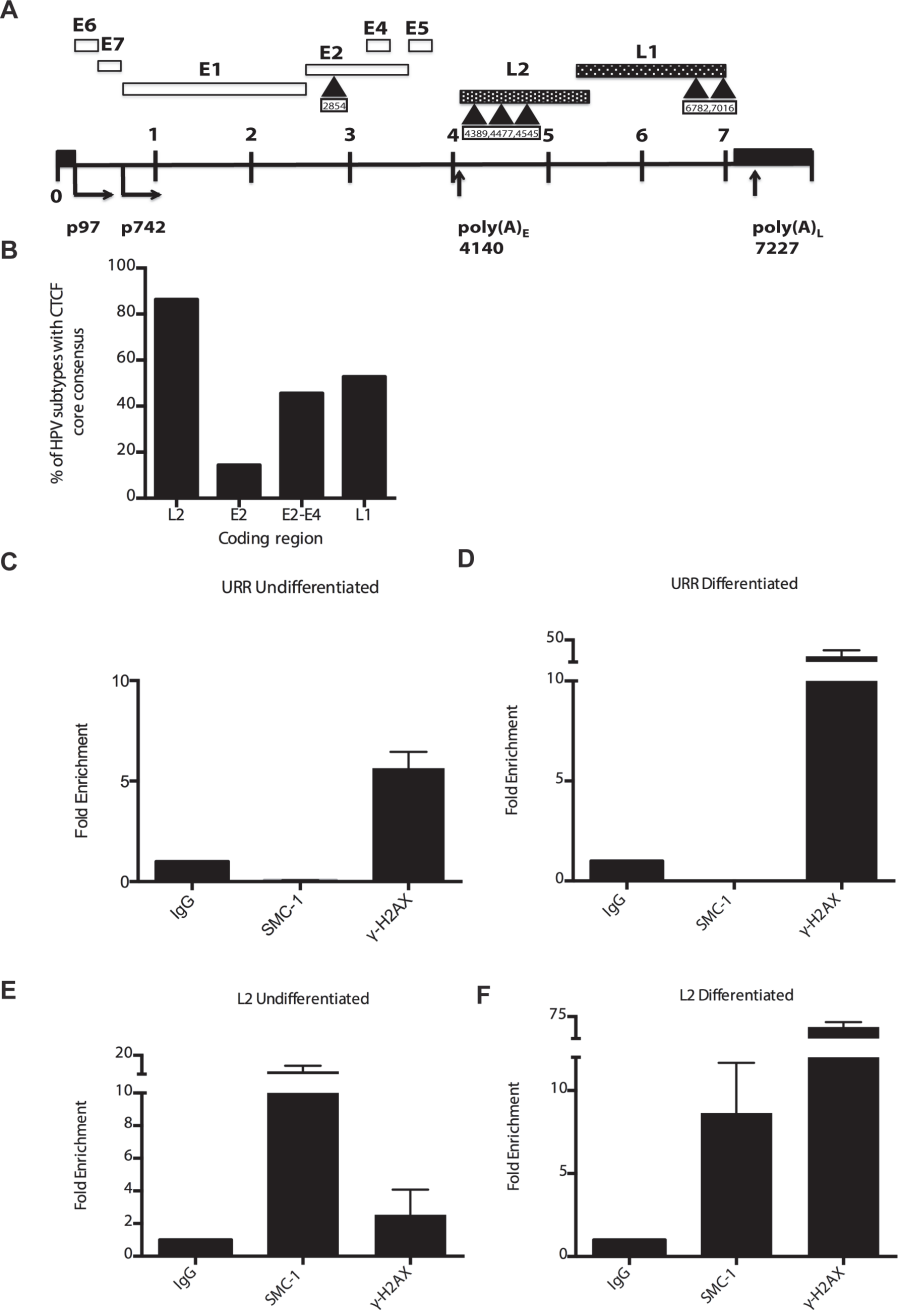
CTCF consensus motifs are found in HPV genomes. A). Schematic diagram of linear HPV 31 genome showing the location of the CTCF (CCCTC) consensus motifs that are also present in other high-risk HPV subtypes. Number indicates the location of the beginning of the pentamer sequence. B). Summary of analysis of 125 HPV types for the presence of CTCF core consensus motifs in L2, L1, E2 and E4 regions. Percentage of HPV type that are positive for these sequences is shown. (C,D). Chromatin immunoprecipitation analysis of SMC1 and γ-H2AX binding to URR region in undifferentiated cells and cells differentiated in calcium for 72 hours. (E,F) Chromatin immunoprecipitation analysis of SMC1 and γ-H2AX binding to the L2 region in undifferentiated cells and cells differentiated in high calcium media for 72 hours. SMC1 binds to the L2 region at similar levels in both undifferentiated or differentiated cells. γ-H2AX binding to HPV genomes increases upon differentiation at both URR and L2 regions Quantitative real-time PCR was performed using a Lightcycler 480 (Roche). Similar results were seen in three independent experiments.

CTCF binds consensus motifs (CCCTC) and we identified similar sequences in the L2 and L1 open reading frames of HPV 31 as well as a potential non-consensus motif in the E2 ORF (Fig 8A). Examination of other HPV types identified similar sequence motifs in the L2 ORFs of over 85% of types while the rest contain CTCF motifs in E2, E4 or L1 (Fig 8B). Additional CTCF sites may also be present that do not consist of canonical CCCTC motifs. In order to identify which regions of the HPV genome were bound by SMC1 proteins, a series of chromatin immunoprecipitation analyses (ChIP) were performed using HPV positive cells. In this analysis we screened for binding to sequences around the origin or replication in the HPV 31 URR as well as sequences around the canonical CTCF binding sites in the L2 open reading frame. Little or no binding of SMC1 was detected at the URR sequences in either undifferentiated or differentiated cells (Fig 8C and 8D). In contrast, significant binding was detected to the L2 region in undifferentiated cells and similar levels were found upon differentiation (Fig 8E and 8F). In addition, we observed low level binding of γ-H2AX to both URR and L2 regions in undifferentiated cells and this increased significantly at both regions upon differentiation (Fig 8C, 8D, 8E and 8F). This is consistent with the reported broad distribution of γ-H2AX binding around sites of DNA damage and is in agreement with our previous report of increased binding of γ-H2AX to viral sequences upon differentiation [16]. An analysis of SMC1 and γ-H2AX binding to HPV sequences as a percentage of input is shown in S3 Fig and is consistent with our analyses using fold binding over IgG (Fig 8). We conclude that SMC1 preferentially binds to the late region of HPV genomes and this is consistent with a role for CTCF in recruiting SMC1 to viral DNAs.

**Fig 9:**
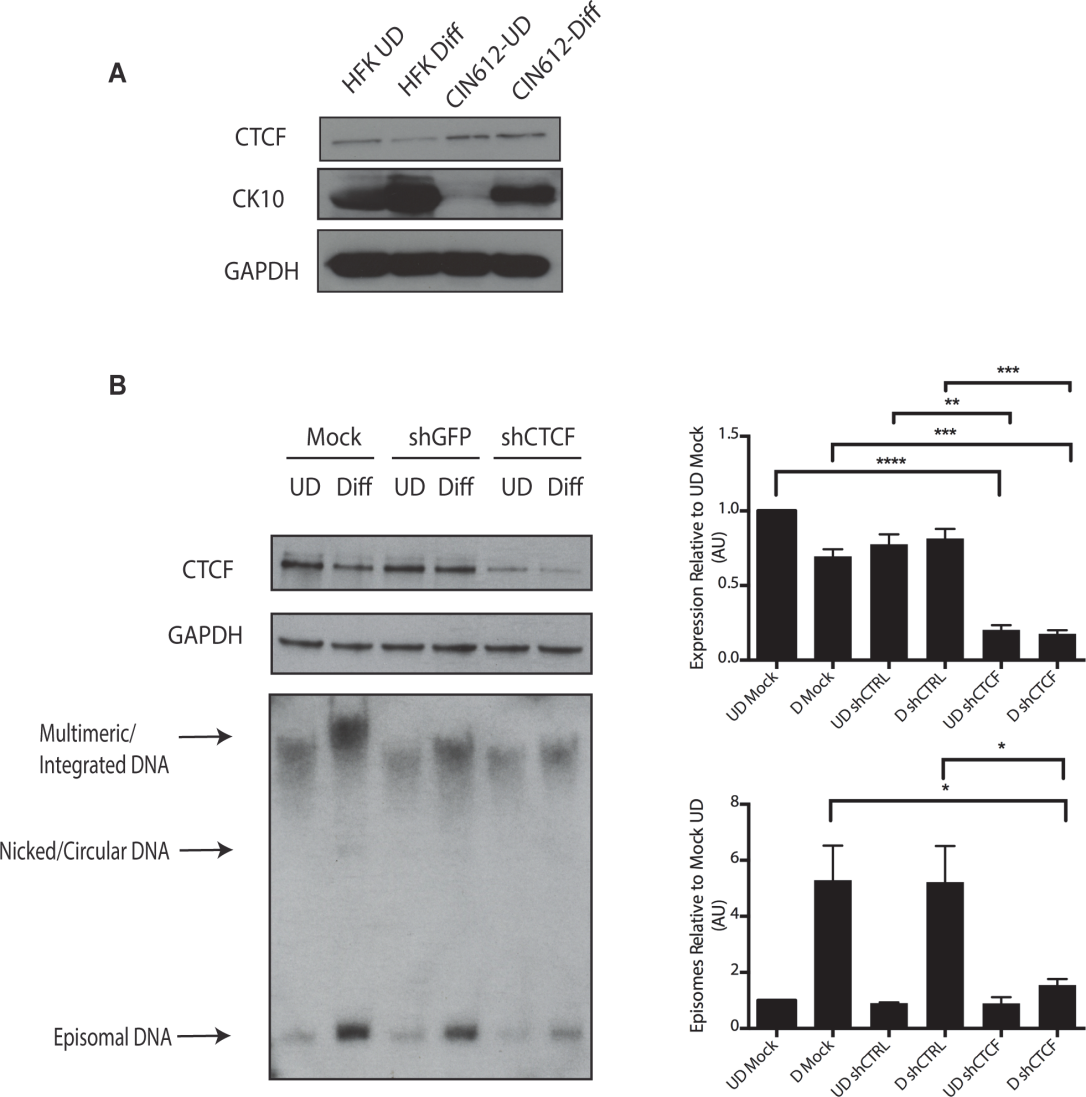
Levels of CTCF are increased in undifferentiated and differentiated HPV positive cells. A). Whole cell extracts were isolated from undifferentiated normal human foreskin keratinocytes (HFK) and CIN 612 cells grown in monolayer culture and examined by Western blot analysis with antibodies to CTCF. Cytokeratin 10 served as a differentiation control. GAPDH served as a loading control. CTCF knockdown results in a statistically significant reduction in protein levels, p≤.01. B). Knockdown of CTCF with shRNA blocks viral genome amplification. CIN612 were infected with lentiviruses encoding shRNA to CTCF and after 48 hours total cell extracts were isolated and analyzed by Western blot for levels of CTCF. shRNA knockdown of CTCF blocks HPV-31 differentiation-dependent viral amplification as shown by Southern blot. Southern blot analysis of CIN 612 cells infected with lentiviruses encoding shRNAs to CTCF and induced to differentiate in methylcellulose for 48 hours. Total DNA was isolated and examined by Southern analysis for HPV amplification. Similar results were observed in three independent experiments using two different shRNAs against CTCF. Quantification of band intensities represents averages of three independent experiments and were determined by densitometry using FIJI software. CTCF knockdown results in a reduction in amplification, p≤.05.

It was next important to examine if CTCF played a role in genome amplification. The levels of CTCF were first examined by Western blot analysis of undifferentiated and differentiated HPV positive cells and compared to those seen in normal HFKs. While CTCF levels were observed to decline in HFKs upon differentiation, they were retained at higher levels in differentiated HPV positive cells (Fig 9A). To determine if CTCF played a role in genome amplification, HPV positive CIN 612 cells were infected with lentiviruses expressing shRNAs against CTCF and screened for levels of viral episomes following differentiation by Southern blot analysis. Since CTCF is an essential gene, we were only able to transiently reduce CTCF levels by approximately 50% as measured by Western blot analysis (Fig 9B). No significant change in the levels of pSMC1 was detected upon reduction in CTCF levels (S4 Fig), In addition, reduced binding of SMC1 to viral genomes was detected by ChIP analysis following CTCF knockdown (S5 Fig) Importantly, we observed a corresponding inhibition of genome amplification when CTCF levels were reduced. Similar results were seen in three independent experiments using two different shRNA vectors as well as the combination of both. CTCF can provide a number of functions including transcriptional regulation and so we performed Northern analysis of HPV transcripts upon CTCF knockdown and found a reduction in levels (S6 Fig) indicating that CTCF acts in multiple ways to regulate the HPV life cycle. Interestingly, reduction of CTCF levels did not alter the ability of γ-H2AX to form foci in HPV positive cells (S7 Fig).

**Fig 10:**
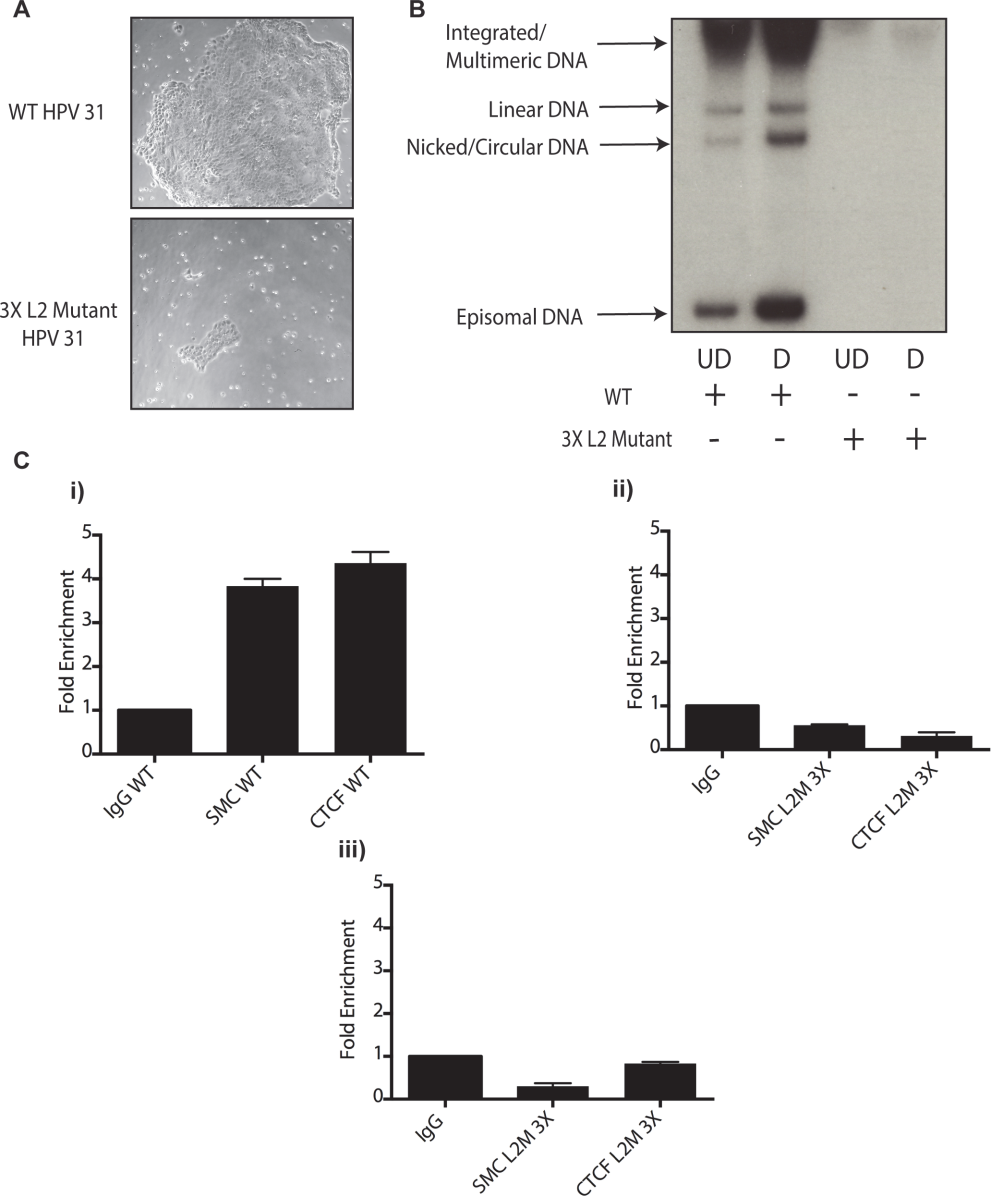
Mutation of 3 CTCF core consensus sequences within the L2 coding region of the HPV 31 genome results in impaired growth, loss of episomes, and rapid integration into the host genome. A).HFKs were transfected with WT HPV-31 genomes or HPV-31 genomes harboring mutations within three CTCF motifs in the L2 coding region along with a drug selectable marker. Following transfection cells were selected and representative colony sizes as seen by light microscopy are shown three days after initiation of selection. Differences in colony size were reproducibly seen. B). Mutation of CTCF sites impairs genome maintenance resulting in integration and inability to amplify. Following transfection and selection, cells were grown to confluency and differentiated in high calcium media for 72 hours. Total DNA was isolated and examined by Southern blot analysis for HPV amplification. Quantification of band intensity was determined by densitometry using FIJI software. C). Mutation of 3 CTCF core consensus sequences abrogates SMC1 and CTCF binding to the HPV genome. Chromatin Immunoprecipitation analysis of stable cell lines containing the WT HPV-31 genome and the three mutations in CTCF motifs demonstrated a reduction of SMC and CTCF binding in mutant lines. Two separate passages of the mutant cell lines demonstrates loss of SMC-1 and CTCF binding. Data is representative of three independent experiments. Panel i). L2 region in cells with wildtype HPV 31; ii). L2 region in cells with L2 mutant 3X genomes at passage 2 after transfection; iii). L2 region in cells with L2 mutant 3X genomes at passage 3 after transfection.

These studies implicate CTCF as an important factor regulating genome amplification. To confirm that CTCF/SMC1 binding was critical to genome amplification, we mutated the three canonical CTCF binding sites in L2 in the context of complete HPV genomes so as to retain the L2 coding sequence intact. The mutant genomes were then sequenced to insure no other mutations were present in the viral DNAs. The mutant viral DNAs as well as wild type genomes were excised from bacterial sequences, religated and transfected into normal human foreskin keratinocytes along with a drug selectable marker. Drug resistant colonies were selected and expanded. In multiple independent transfections we observed that colonies containing mutant genomes grew more slowly than wild type transfected cells (Fig 10A). The colonies were expanded and the state of viral DNA was examined by Southern blot analysis. Cells with wildtype genomes exhibited high levels of episomal DNAs that were stably maintained and amplified upon differentiation. In contrast, cells containing CTCF mutant genomes contained very low levels of episomes that were rapidly lost with passage. The cells with mutant genomes contained primarily integrated copies and failed to amplify upon differentiation (Fig 10B). This suggests that the CTCF binding sites in L2 play a central role in the HPV life cycle including an unexpected potential role in stable maintenance of episomes. Finally, it was important to confirm that the mutations introduced into HPV 31 genomes abrogated the binding of CTCF and SMC1 proteins. ChIP analysis was performed on the wild type and mutant genome containing cells and no binding of CTCF or SMC1 to the L2 region was detected at both an early passage following transfection as well as in subsequent passages (Fig 10C and S3 Fig). In contrast, both factors were found to bind to L2 in cells containing wild type viral genomes. Interestingly, pSMC1 still formed foci in cells with integrated HPV genomes though they appear localized to only one or two foci (S7 Fig). These studies demonstrate that CTCF and SMC1 binding to the L2 region is critical for genome amplification as well as a identifying a novel role in stable genome maintenance.

## Discussion

Our studies demonstrate that the cohesin protein, SMC1, is a critical factor in regulating HPV genome amplification in differentiating cells. SMC1 is a member of the cohesin complex that mediates sister chromatid cohesion, which is necessary for proper chromosomal segregation in mitosis [35]. The phosphorylated form of SMC1 also functions in the ATM DNA damage repair pathway and facilitates homologous recombination repair [24],[23]. Our studies indicate that HPV genomes increase the levels of total and phosphorylated SMC1 in the absence of irradiation or other DNA damaging agents. This contrasts with what occurs in normal cells where exposure to irradiation or other damaging agents is required for induction of SMC1 phosphorylation[24]. The constitutive activation of SMC1 phosphorylation in HPV positive cells suggests it has an important role in the viral life cycle.

The activation of the ATM DNA damage pathway is necessary for HPV genome amplification upon differentiation but it has little effect on transient or stable maintenance replication in undifferentiated cells [19]. Previous studies indicated that HPV genome amplification is dependent on the activities of the CHK2 arm of the ATM pathway, however, it was unclear if the NBS1/SMC1 factors, which form a separate and independent arm of the ATM pathway, played any role[19]. Both arms act independently and both mediate cell cycle arrest in late S/G2, which this is consistent with the timing of HPV genome amplification [9]. In our studies, the partial knockdown of SMC1 was sufficient to block genome amplification upon differentiation and indicates that both the CHK2 and SMC1/NBS1 arms of the ATM pathway provide essential activities. This is further supported by our observations that pSMC1 forms complexes with γ-H2AX and CHK2 in HPV positive cells together forming replication centers associated with the viral DNA. Recent studies have also confirmed the presence of NBS1 at HPV-31 viral foci and its importance in HPV 31 genome amplification [36].

Proximity ligation analyses together with chromatin immunoprecipitation experiments indicate that SMC1 is recruited to HPV genomes through complex formation with CTCF insulator proteins. SMC1 is preferentially recruited to the L2 region of HPV 31 that contains three canonical CTCF binding sites. In normal cells, SMC1 forms complexes with only a subset of CTCF proteins and CTCF can bind DNA in the absence of cohesins[37], [38]. In our studies, we identified CTCF sequence motifs in L2, L1 and a potential site in E2. The three motifs in L2 are canonical sites that are separated by spacing typically seen in active CTCF sites[39]. Chromatin immunoprecipitation analyses demonstrated similar levels of SMC1 binding to the L2 region in undifferentiated and differentiated cells while the levels of γ-H2AX binding increased. Importantly, mutation of these sites results in abrogation of binding of both SMC1 and CTCF and viral genomes containing these mutations quickly lose the ability to be stably maintained as episomes as well as to amplify upon differentiation. Finally, transient knockdown of CTCF with shRNAs inhibited amplification and transcript levels. Since we were not able to establish cells in which CTCF is stably knocked down, we were unable to screen these cells for an effect on long-term stable maintenance of episomes. Overall, these findings implicate SMC1 interactions with CTCF as critical for amplification of HPV genome upon differentiation.

The inability of genomes with mutated CTCF sites to be stably maintained as episomes in undifferentiated cells was unexpected and indicates that CTCF may have roles in the HPV life cycle beyond recruiting pSMC1 to viral DNAs as part of the ATM DNA damage response. CTCF complexes mediate insulator function by blocking adjacent enhancer function and CTCF complexes bound to HPV genomes may also act as insulator elements that shield cryptic viral promoters from activation by the HPV enhancer element in the URR[34], [29]. This could provide specificity to the activation of the HPV enhancer, permitting only unidirectional transcription from early and late promoters around the E6 and E7 ORFs. In support of a role of CTCF in regulating HPV transcription, we observed reduced levels of HPV early transcripts in CTCF knockdown cells. CTCF has also been shown to play a role in regulating chromatin assembly, gene regulation and looping in herpesviruses[40]. The Lieberman group has shown that in KSHV and EBV, CTCF is responsible for organizing chromatin loops that regulate latent gene expression and mutation of CTCF binding sites reduces stable genome copy number[41],[42]. It is likely that CTCF functions to organize intramolecular loops on HPV genomes in a similar manner. These loops could form between different CTCF sites as well as potentially with E2 factors bound to URR sequences. These sites could be located on the same genome or on nearby sister genomes. The E2 proteins have been shown to form complexes with ChlR1 to promote proper viral genome segregation. ChlR1 has also been shown to bind SMC1 as well as other members of the cohesin complex [43]. This suggests that CTCF’s role in the stable maintenance of HPV episomes could be through its association with SMC1 and tethering factors such as ChlR1. In this way intermolecular looping with other viral genomes as well as cellular CTCF sites may occur. DNA looping mediated by CTCF/cohesion complexes is a dynamic process that changes through the cell cycle, and could play a role in modulating the structure of HPV genomes during the differentiation dependent life cycle. Further analysis of looping of viral DNAs can shed light on the role CTCF/cohesin complexes in genome maintenance.

Our studies suggest that the role of SMC1/CTCF complexes in HPV genome amplification is part of the ATM-dependent homologous recombination repair pathway. In normal cells homologous recombination repair of DNA occurs in G2 after chromosomes have replicated and sister chromatids are brought into proximity through the action of cohesins. In HPV positive cells, SMC1 cohesin complexes may be functioning in a similar manner during amplification by aligning multiple HPV genomes together in G2. HPV amplification has been shown to be dependent on homologous DNA repair factors such as RAD51 and BRCA1[20]. In addition, amplification occurs in G2, which is when homologous recombination occurs[6]. SMC1 is also a component of the RC-1 homologous recombination complex that contains single strand binding protein RPA and RAD51, as well as the repair polymerase ε and DNA ligase III [18]. It is possible that SMC1 helps to recruit homologous repair polymerases to viral genomes to mediate replication of viral genomes during the amplification process. Repair polymerases such as DNA polymerase epsilon and ligases such as DNA ligase III could be the principal mediators of viral amplification rather than polymerase α and polymerase δ that replicate host chromosome DNAs in early to mid S-phases. Since HPV genomes are only approximately 8 kilobases in size, replication could easily be accomplished by repair polymerases.

In normal cells, SMC1 proteins are bound at discrete sites on chromosomes and upon DNA damage they become phosphorylated and are recruited to sites of double strand breaks. It is possible that double strand breaks are generated in HPV genomes prior to or during amplification and that SMC1 cohesin protein complexes migrate from the sites in L2 to double strand breaks. The mechanism responsible for the generation of double strand breaks in HPV genomes is unclear and to date we have no evidence that double strand breaks are present in HPV genomes. If these sites exist and are generated in a random fashion, then SMC1 binding might not exhibit sequence specificity other than to the L2 sites. If double strand breaks are generated at distinct sites during HPV genome amplification, then there should be specificity of recruitment of SMC1 and other ATM factors to these sites that should be detected by Southern analysis. A rolling circle model of replication has been suggested for HPV genome amplification and this would require site-specific cleavage of viral DNA concatamers[44]. This model however remains controversial and it is equally possible that HPV genomes amplify through theta structure replication. In theta replication the resolution of interlocked replicative intermediates would also require the generation of double strand breaks during cleavage/religation of circular intermediates and such an activity has been suggested for ATMs role in SV40 replication[45]. This mechanism should result in the formation of large replicative structures upon addition of ATM inhibitors but we have not observed such complexes of HPV genomes in our preliminary studies. Further understanding of the mechanism by which homologous recombination factors together with SMC1 and CTCF contribute to HPV genome amplification will require a detailed structural analysis of viral DNAs undergoing amplification. During the submission of this study, we became aware of a study from the Parish lab (Paris et al.) that identifies CTCF sites in HPV 18 and demonstrates CTCF to be a critical regulator of HPV 18 gene expression and splicing. These studies are in agreement with our work and demonstrate that CTCF is an important regulator of HPV biology. Overall, our studies identify SMC1 and CTCF as critical regulators of viral replication during the differentiation-dependent life cycle of human papillomaviruses.

## Materials and Methods

### Ethics statement

The human keratinocytes used in this study were obtained from discarded foreskin circumcisions from anonymous donors by the Keratinocyte Core in the Northwestern University Skin Disease Research Center (SDRC) and are not classified as human subjects research. These specimens were not specifically collected for this study and lack all identifiers.

### Cell culture

Human foreskin keratinocytes (HFKs) were isolated from neonatal human epidermis. HFKs containing HPV genomes were cultured in E-medium supplemented with mouse epidermal growth factor (EGF) (Becton Dickenson, Franklin Lakes, New Jersey, Cat. #354010). The HFKs were cultured on J2 fibroblasts arrested with mitomycin-c as previously described[46]. CIN-612 cells, which maintain the HPV-31 viral genome episomally were cultured in E-medium + EGF, with mitomycin-c treated J2 feeders.

### Plasmids

pBR-322min containing HPV 31 was used for the generation of HPV 31 cell lines (HFK-31) along with pSV-Neo2 a neomycin resistance plasmid and used for stable cell line selection. Validated shRNA constructs in the pLKO.1 lentiviral background targeting SMC1 and CTCF were purchased from Sigma-Aldrich (St Louis, Missouri) as was pLKO.1 shGFP. pVSVG and pGag-Pol-Tat-Rev were used for the generation of lentiviral particles.

### Generation of cell lines stably transfected with HPV31

Recircularized HPV-31 was created by the removal of the pBR-322 backbone from pBR-322min-HPV31 and subsequent religation. Recircularized HPV-31 genomes were contransfected with a neomycin resistance expression vector using FuGene-6 (Roche), after versene treatment to remove J2 fibroblast feeders. One million cells were seeded on each 100-mm dish and transfected with plasmid DNA once at 60% confluency. After 24 hours, cells were selected with G418 and expanded as previously described[47].

### Generation of HPV-31 L2 mutant

The three CTCF consensus sequences within the L2 coding region of the HPV-31 genome (pBR-322) were mutated using site-directed mutagenesis so as to maintain L2 coding sequence intact and verified by sequencing (Clontech, Mountain View, California, R045A). Site one starting at nucleotide 4387 5’-GTC|CCT|CTT-3’ was changed to 5’-GTC|CCG|CTT-3’. Site two starting at nucleotide 4477 5’-CCC|TCT|-3’ was changed to 5’-CCA|AGC|-3’. Site three starting at nucleotide 4543 5’-CAC|CCT|CCT|AC-3’ was changed to 5’-CAT|CCG|CCG|AC-3’. Stable cell lines containing the WT or the 3X L2 mutant were transfected into the same HFK background and stably selected as described above.

### Calcium induced differentiation

To induce differentiation, HPV negative and positive HFKs as well as CIN-612 cells were switched to Invitrogen’s M154 with HKGS and Pen-Strep and. 03mM filter-sterilized calcium chloride for 24 hours and allowed to grow to 80% confluency. The media was changed to Invitrogen’s M154 without HKGS plus Pen-Strep and 1.5mM filter-sterilized calcium chloride, and cultured for 72 hours to induce differentiation as previously described [48].

### Methylcellulose induced differentiation

HPV negative and positive HFKs as well as CIN-612 cells were cultured as outlined above. To induce differentiation, 3 million cells were harvested following versene treatment to remove J2 fibroblast feeders. The cells were then resuspended in 1.5% methylcellulose that was dissolved in E-media. Following resuspension, the cells were allowed to grow for 48 hours. They were subsequently harvested for protein lysates or total genomic DNA as previously described [49].

### Western blot analysis

Whole cell lysates were extracted using RIPA lysis buffer, following versene treatment for the removal of J2 3T3 feeder fibroblasts. Protein was quantitated using the Bio-Rad (Hercules, California) bradford assay and 40 g of protein was loaded per well, and run on 8.0%, 10.0%, or 4–20% SDS-polyacrylamide gels. The gels were wet transferred to Immoblion-P, PVDF (Millipore, Billerica, Massachusetts) and probed with the primary and secondary antibodies. ECL (GE, Pittsburgh, Pennsylvania) was used as the chemiluminescent substrate.

### Antibodies

Phospho-CHK2-Thr-68 (Cell Signaling, Danver, Massachusetts, Cat. #2661), CHK2 (Cell Signaling, Cat. #3440), Phospho-SMC1 (S957) (Abcam, Cambridge, Massachusetts, Cell Signaling, Cat. #ab1275, 4805, Bethyl, Cat. #A304-147A-M, Montgomery, Texas), SMC1 (Abcam, Cell Signaling Cat #ab9262, 4802), CTCF (BD, Franklin Lakes, New Jersey, Cell Signaling, Millipore Cat #612149, 2899, 07–729, 17–10044), γ-H2AX (Millipore, Cell Signaling, Cat #05–636, 5438), GAPDH (Santa Cruz, Paso Robles, California).

### Immunofluoresence

Cells were seeded at subconfluence onto coverslips (No. 1). After 24 hours, the cells were treated with 4% Methanol-free formaldehyde in PBS, differentiated in calcium as described above. Next, the cells were permeabilized in PBS + 0.1% Triton X-100 (PBT) and blocked in Normal Goat Serum (NGS+T) (Invitrogen) with 0.1% Triton X-100. Primary antibodies were added to the NGS+T according to manufacturer’s specifications or at 1:50 if not specified. Washes were performed in PBST. Secondary antibodies (Alexa Fluor, Invitrogen) were added to NGS+T. All incubations were performed in a humidity chamber. Slides were mounted using Pro-Long Gold Antifade + DAPI (Invitrogen), or Gelvatol (homemade) and DAPI and imaged using a Zeiss Axioscope. Image analysis performed in Axiovision and Fiji Is Just Image J (FIJI).

### Proximity ligation assays

Cells were seeded at subconfluence on coverslips (No. 1). All cell-types were seeded without J2 fibroblast feeders. After 24 hours, the cells were treated with 4% Methanol-free formaldehyde in PBS. Subsequently the cells were permeabilized in PBS + 0.1% Triton X-100 (PBST) and blocked in Normal Goat Serum (NGS+T) (Invitrogen) with 0.1% Triton X-100. Primary antibodies were added to the NGS+T according to manufacturer’s specifications or at 1:50 if not specified. Washes were performed in PBST. Secondary antibodies, covalently linked with plus/minus oligos were diluted in NGS + T as per the manufacturer’s instructions (OLINK DuoLink, Upsalla Sweden). Ligations, and polymerase amplifications, and washes were performed as per the manufacturer’s instructions. All incubations were performed in a humidity chamber.

### Chromatin immunoprecipitation

Chromatin immunoprecipitations were performed using CIN612 or stable HPV-31 (WT or L2 Mutant 3X) cells grown as described above. Before fixation, cells were treated with versene to remove J2 fibroblast feeders. Cells were then seeded and grown to 80% confluency. Chromatin-immunoprecipitation was performed as previously described using SMC1 (Abcam, Cat. #ab9262), γ-H2AX (Millipore Cat. #05–636), CTCF (Millipore, Cat. #07–729), Normal Rabbit IgG (Santa Cruz, Cat. #SC-2027), Normal Mouse IgG Antibody (Santa Cruz, Cat. #SC-2025), Normal Mouse IgG (Millipore, Cat. #12-371B) and Protein-G Dynabeads (Invitrogen Cat. #100-03D) [50]. Real-time, touchdown PCR was performed with the Lightcycler 480 (Roche) and the HPV-31 CTCF Trio Primers and URR Primers; Forward: 5’-TTTGGTGGGTTGGGTATTGG-3’, Reverse:5’-GTAGGAGGCTGCAATACAGATG-3’. Forward, 5’-AACTGCCAAGGTTGTGTCATGC 3’, Reverse, 5’-TGGCGTCTGTAGGTTTGCAC-3’.

### Lentiviral knockdowns

Two MISSION pLKO.1 shRNA constructs for SMC1 (Sigma), two constructs targeting CTCF, or shGFP (shCTRL) were transfected (3g) into 50% confluent 293T cells along with pVSVG and pGag-Pol-Tat-Rev via X-tremeGENE HP DNA Transfection Reagent (Roche, Indianapolis, Indiana, Cat. #06366236001). 24 hours post-transfection, the media was changed and the cells were allowed to grow for another 24 hours. The viral supernatants were collected, and concentrated using a Millipore concentrator (Cat. #UFC910096). For infections, 2 million cells were seeded and allowed to grow for twenty-four hours. The concentrated viral particles were either pooled or singly distributed equally to cells in the presence of 0.8 g /ml Polybrene (Sigma-Aldrich) in 3 mls of media. After 6 hours, an additional 4 mls of media were added. Twenty-four hours post-transduction, the media was changed, and the cells were allowed to grow for another 24 hours. Cells were then either harvested or differentiated using methylcellulose. Knockdown was confirmed using western blot analysis.

### KU60019 and caffeine inhibition of pSMC1 S957

CIN612 cells were treated with or without ATM-kinase inhibitor KU-60019 (10uM, Tocris, Bristol, UK, Cat. #4176) or Caffeine (100mM Stock) (Sigma, Cat. # C0750) or their respective vehicle (DMSO, PBS) in E-medium supplemented with mouse epidermal growth factor (EGF) for the indicated times as previously described [26],[51]. The cells were subsequently harvested for protein.

### Southern and northern blot analysis

CIN-612 or HPV-31 (WT or L2 Mutant 3X) cells were treated with versene to remove J2 3T3 feeder fibroblasts. Southern blot lysis buffer (400 mM NaCl, 10 mM Tris-HCl, 10 mM EDTA) was used to induce lysis of undifferentiated or calcium or methycellulose-differentiated cells. Cells were subsequently treated with RNase A (Sigma-Aldrich) and proteinase K (Sigma-Aldrich). Total DNA was isolated by phenol-chloroform extraction, and Southern analysis was carried out as previously described[52]. Total RNA was collected in STAT-60 and isolated by phenol-chloroform extraction. Northern analysis was carried out as previously described [52].

## Supporting Information

S1 Fig Levels of total SMC1 and pSMC1 are increased in HFKs expressing HPV-31 E7. Whole cell extracts were isolated from undifferentiated normal human foreskin keratinocytes (HFK) stably transduced and selected following infection with retroviruses expressing HPV-31 E7 grown in monolayer culture. Cell lysates examined by Western blot analysis with antibodies to total SMC1, pSMC1. GAPDH served as a loading control. Levels of p53 shown as an indicator of E7 expression.
(TIF)

S2 Fig Proliferation and viability assays of SMC-1 knock down cells. CIN612 cells were plated at a density of one million cells per plate and transduced the next day with lentiviruses expressing shRNAs to SMC1 or shGFP as a control. Mock cells served as a non-transduction control. After 48 hours of transduction, cells were trypsinized, stained with trypan blue and were counted for total number of cells to determine proliferation. Cells were also stained for live/dead cell ratio to determine viability assay. A). The proliferation assay is represented as total cell number in millions at 0 hour and 48 hours post transduction. B.) The cell viability assay is represented as percentage viability which is the percentage of live cells out of the total cells at 48 hours post transduction. No significant difference was observed in both proliferation and viability of SMC-1 knock down cells compared to control cells. Data is an average of four independent experiments.
(TIF)

S3 Fig Chromatin immunoprecipitation analysis from Fig 9A–9D and Fig 10 (E) represented as percent input. Multiple IgGs are represented due to different hosts in which the primary antibodies were made. See Figure legends for details.
(TIF)

S4 Fig Levels of total SMC1 and pSMC1 are not affected by shRNA knockdown of CTCF. A.) Whole cell extracts were isolated from Mock, shCTRL transduced, and shCTCF transduced cells. Lysates were examined by Western Blot analysis with antibodies to total SMC1, pSMC1, and CTCF. GAPDH served as a loading control.
(TIF)

S5 Fig Knockdown of CTCF with shRNA leads to a reduction in SMC1 occupancy at the L2 coding region as shown by chromatin-immunoprecipitation analysis. Quantitative real-time PCR was performed using a Lightcycler 480 (Roche). Chromatin immunoprecipitation data is shown both as fold enrichment over IgG and percent input.
(TIF)

S6 Fig Knockdown of CTCF with shRNA leads to a reduction in HPV early transcripts. A.) CIN612 cells were infected with lentiviruses encoding shRNAs to CTCF and after 48 hours total RNA was isolated and analyzed by Northern blot for levels of HPV early transcripts (E6/E7/E1^E4/E5 and E6*/E7/E1^E4/E5).
(TIF)

S7 Fig Introduction of the 3X L2 mutant or knockdown of CTCF with shRNA still induces formation of pSMC1 or γ-H2AX. A.) WT 31 and 3X L2 mutant cells were differentiated in calcium for 72 hours and analyzed using immunofluorescence. Green represent pSMC1, and blue nuclear DAPI staining. pSMC1 still formed foci in cells with integrated HPV genomes though they appear localized to one or two foci B.) CIN612 cells were infected with lentiviruses encoding shRNAs to CTCF or control shRNAs. CTCF knockdown was analyzed by western blot (S4 Fig). Cells were allowed to differentiate in calcium for 72 hours and were analyzed using immunofluorescence. Green represents pSMC1, red represents γ-H2AX, and blue represent nuclear DAPI staining (merges shown). Quantitation shown represents an n of 82, 51, and 97 for Mock, shCTRL, and shCTCF respectively
(TIF)
