# Supplementary figures and images for: Human Papillomaviruses Activate and Recruit SMC1 Cohesin Proteins for the Differentiation-Dependent Life Cycle through Association with CTCF Insulators
Source: PLoS Pathog. 2015 Apr 13;11(4):e1004763. doi: 10.1371/journal.ppat.1004763 (PMC4395367; doi:10.1371/journal.ppat.1004763)

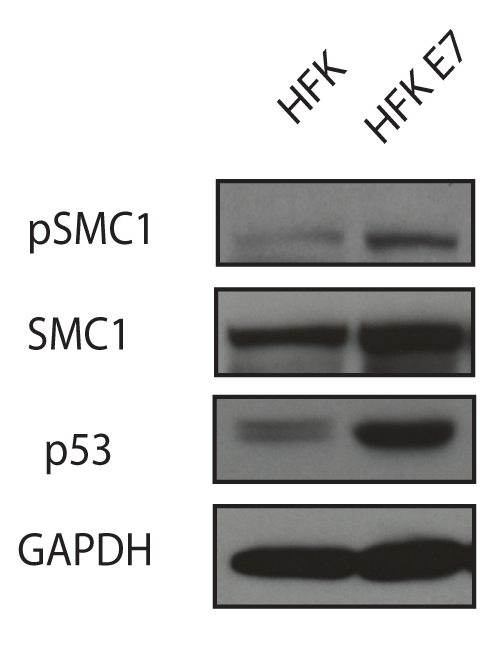

Supplement: S1 Fig — Whole cell extracts were isolated from undifferentiated normal human foreskin keratinocytes (HFK) stably transduced and selected following infection with retroviruses expressing HPV-31 E7 grown in monolayer culture. Cell lysates examined by Western blot analysis with antibodies to total SMC1, pSMC1. GAPDH served as a loading control. Levels of p53 shown as an indicator of E7 expression. (TIF) [file ppat.1004763.s001.tif]

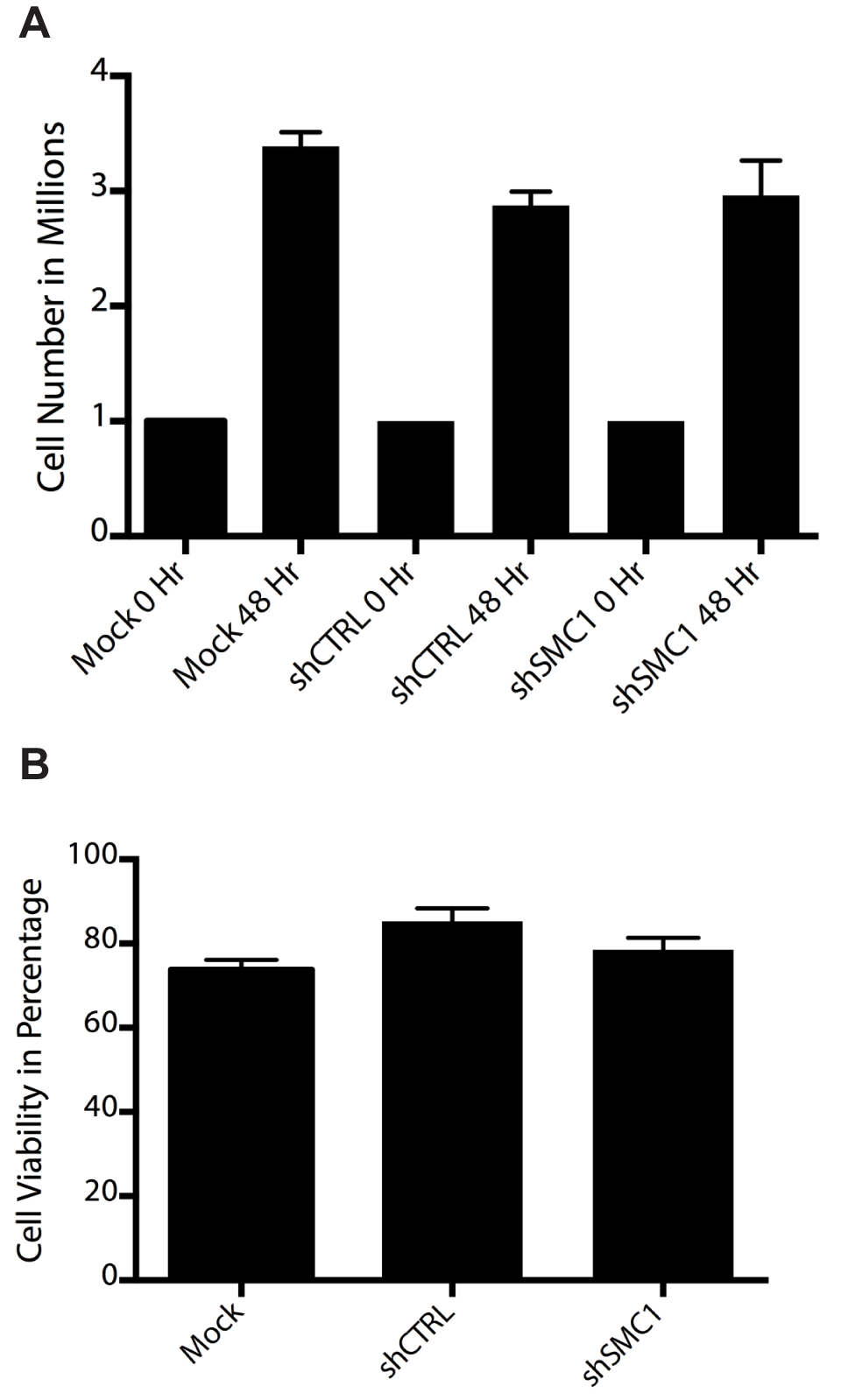

Supplement: S2 Fig — CIN612 cells were plated at a density of one million cells per plate and transduced the next day with lentiviruses expressing shRNAs to SMC1 or shGFP as a control. Mock cells served as a non-transduction control. After 48 hours of transduction, cells were trypsinized, stained with trypan blue and were counted for total number of cells to determine proliferation. Cells were also stained for live/dead cell ratio to determine viability assay. A). The proliferation assay is represented as total cell number in millions at 0 hour and 48 hours post transduction. B.) The cell viability assay is represented as percentage viability which is the percentage of live cells out of the total cells at 48 hours post transduction. No significant difference was observed in both proliferation and viability of SMC-1 knock down cells compared to control cells. Data is an average of four independent experiments. (TIF) [file ppat.1004763.s002.tif]

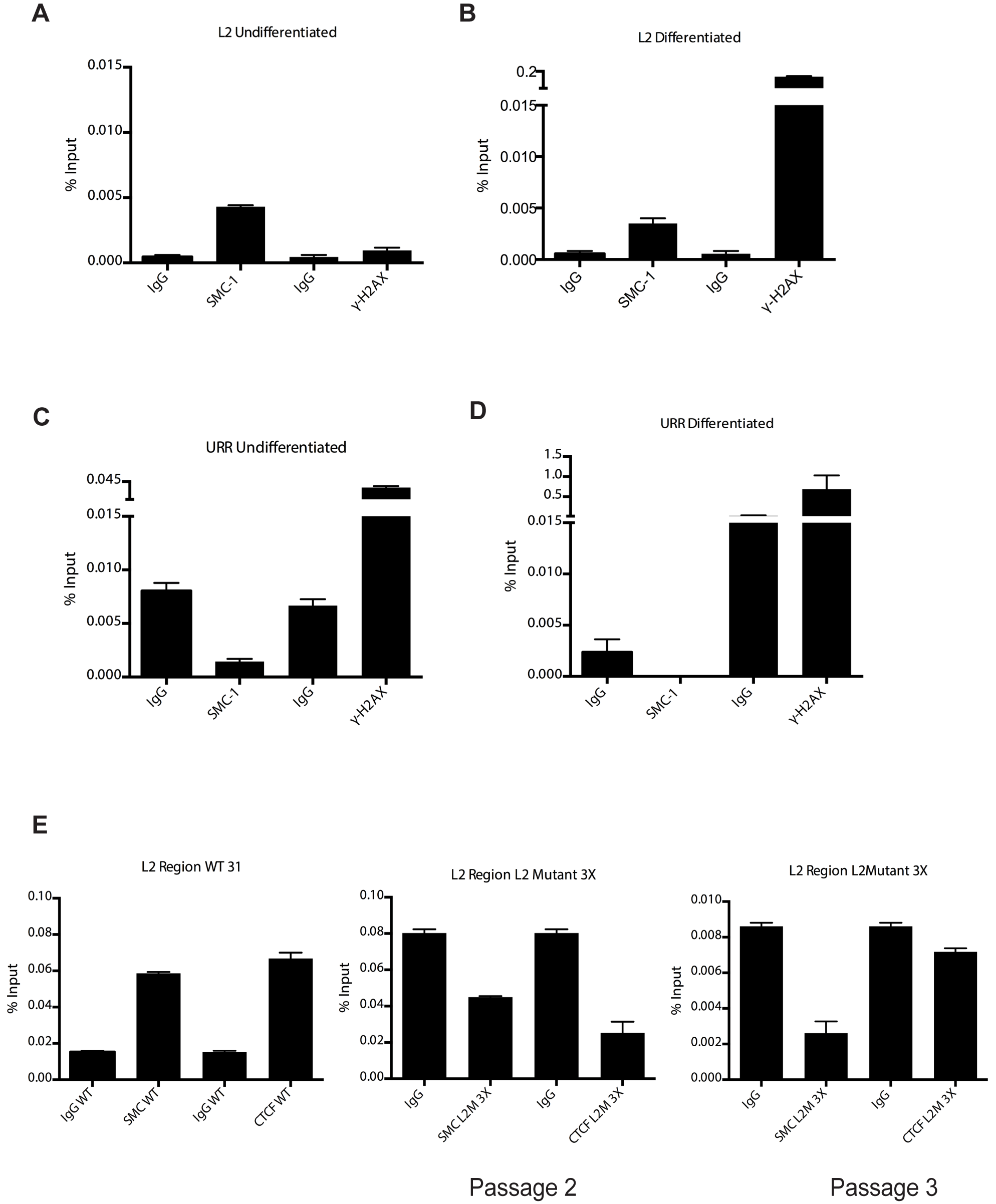

Supplement: S3 Fig — Multiple IgGs are represented due to different hosts in which the primary antibodies were made. See Figure legends for details. (TIF) [file ppat.1004763.s003.tif]

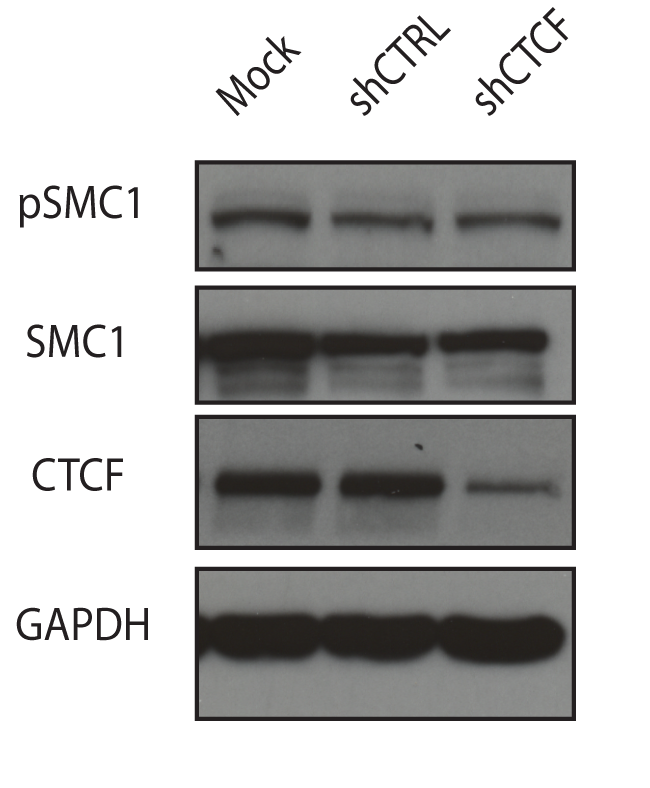

Supplement: S4 Fig — A.) Whole cell extracts were isolated from Mock, shCTRL transduced, and shCTCF transduced cells. Lysates were examined by Western Blot analysis with antibodies to total SMC1, pSMC1, and CTCF. GAPDH served as a loading control. (TIF) [file ppat.1004763.s004.tif]

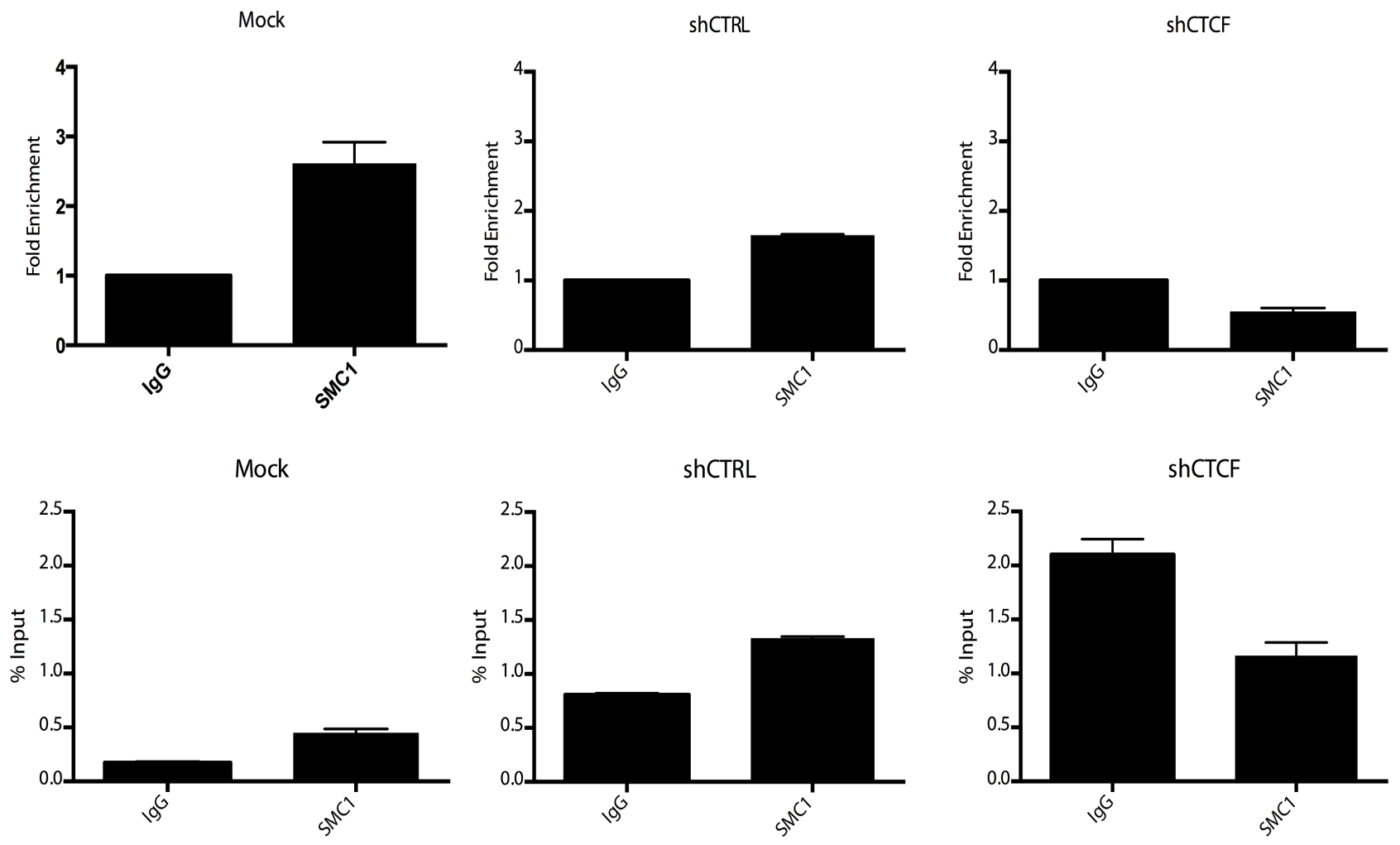

Supplement: S5 Fig — Quantitative real-time PCR was performed using a Lightcycler 480 (Roche). Chromatin immunoprecipitation data is shown both as fold enrichment over IgG and percent input. (TIF) [file ppat.1004763.s005.tif]

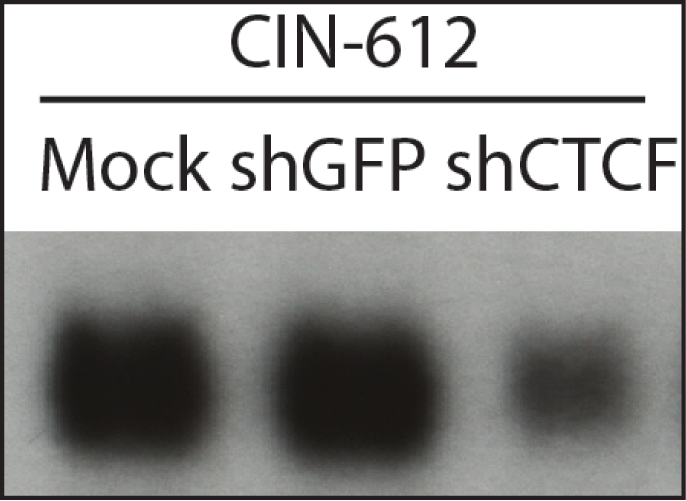

Supplement: S6 Fig — A.) CIN612 cells were infected with lentiviruses encoding shRNAs to CTCF and after 48 hours total RNA was isolated and analyzed by Northern blot for levels of HPV early transcripts (E6/E7/E1^E4/E5 and E6*/E7/E1^E4/E5). (TIF) [file ppat.1004763.s006.tif]

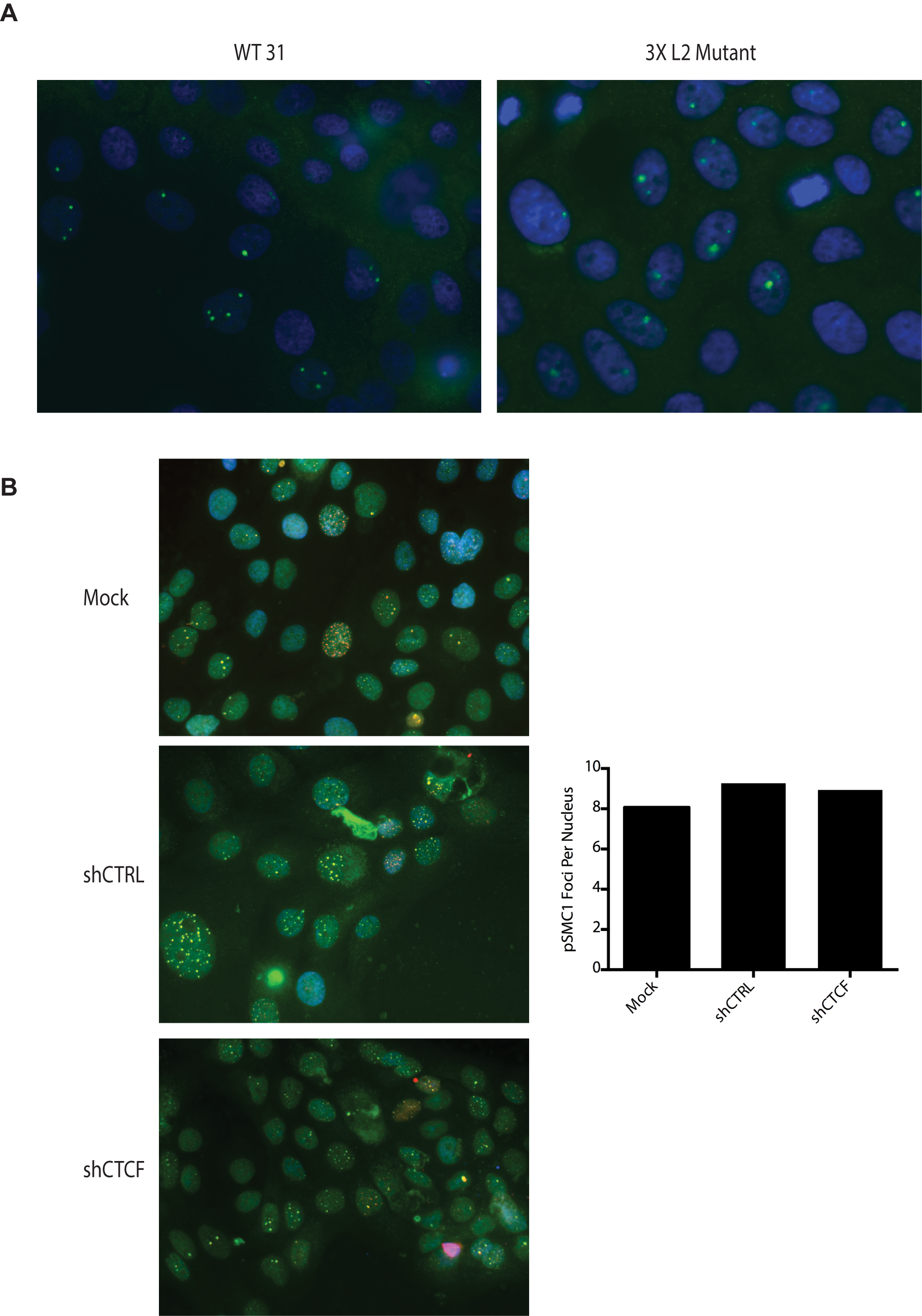

Supplement: S7 Fig — A.) WT 31 and 3X L2 mutant cells were differentiated in calcium for 72 hours and analyzed using immunofluorescence. Green represent pSMC1, and blue nuclear DAPI staining. pSMC1 still formed foci in cells with integrated HPV genomes though they appear localized to one or two foci B.) CIN612 cells were infected with lentiviruses encoding shRNAs to CTCF or control shRNAs. CTCF knockdown was analyzed by western blot (S4 Fig). Cells were allowed to differentiate in calcium for 72 hours and were analyzed using immunofluorescence. Green represents pSMC1, red represents γ-H2AX, and blue represent nuclear DAPI staining (merges shown). Quantitation shown represents an n of 82, 51, and 97 for Mock, shCTRL, and shCTCF respectively (TIF) [file ppat.1004763.s007.tif]
